# A Convolutional Neural Network for SSVEP Identification by Using a Few-Channel EEG

**DOI:** 10.3390/bioengineering11060613

**Published:** 2024-06-15

**Authors:** Xiaodong Li, Shuoheng Yang, Ningbo Fei, Junlin Wang, Wei Huang, Yong Hu

**Affiliations:** 1 Orthopedics Center, The University of Hong Kong-Shenzhen Hospital, Shenzhen 518053, China; 2 Department of Orthopaedics and Traumatology, The University of Hong Kong, Hong Kong SAR, China; 3 Department of Rehabilitation, The Second Affiliated Hospital of Guangdong Medical University, Zhanjiang 524003, China

**Keywords:** steady-state visual evoked potential (SSVEP), brain–computer interface (BCI), electroencephalogram (EEG), convolutional neural network (CNN), few channel

## Abstract

The application of wearable electroencephalogram (EEG) devices is growing in brain–computer interfaces (BCI) owing to their good wearability and portability. Compared with conventional devices, wearable devices typically support fewer EEG channels. Devices with few-channel EEGs have been proven to be available for steady-state visual evoked potential (SSVEP)-based BCI. However, fewer-channel EEGs can cause the BCI performance to decrease. To address this issue, an attention-based complex spectrum–convolutional neural network (atten-CCNN) is proposed in this study, which combines a CNN with a squeeze-and-excitation block and uses the spectrum of the EEG signal as the input. The proposed model was assessed on a wearable 40-class dataset and a public 12-class dataset under subject-independent and subject-dependent conditions. The results show that whether using a three-channel EEG or single-channel EEG for SSVEP identification, atten-CCNN outperformed the baseline models, indicating that the new model can effectively enhance the performance of SSVEP-BCI with few-channel EEGs. Therefore, this SSVEP identification algorithm based on a few-channel EEG is particularly suitable for use with wearable EEG devices.

## 1. Introduction

As a brain–computer interface (BCI) allows the human brain to interact directly with external environment and devices, it has shown great application potential in many fields, such as rehabilitation, sport and entertainment. By decoding electroencephalogram (EEG) signals detected on the scalp, a BCI can transfer human intentions into communication or control demands. For EEG measurement devices, most of them are designed for medical or scientific research purposes. They are generally large in size, heavy and require complex operating procedures, making them unsuitable for daily use in real life. With the advancement of electronic technology, many wearable EEG devices have been designed and produced. Owing to their compact structure, light weight and good wearability, wearable EEG devices have gradually been used in BCI applications, such as robot control [1], remote monitoring [2] and emotion recognition [3]. Compared with conventional EEG devices, wearable EEGs typically support a lower number of channels. Multi-channel data generally achieves better BCI performance, as it contains more information. However, more EEG channels mean a longer preparation time and reduced comfort, which is the opposite of the intention of wearable BCIs. Moreover, reducing the number of electrodes effectively lowers the hardware cost of wearable BCIs. Therefore, few-channel EEGs are an attractive option for wearable BCIs. On the other hand, classification performance is a key factor in BCI systems because it is related to the usability and usefulness of the BCI. For wearable BCIs, it is necessary to use few-channel EEGs to achieve comparable performance to enhance system practicality.

Steady-state visual evoked potential (SSVEP) is a classic BCI paradigm that has garnered substantial attention and has been widely used as it supports multiple instructions, achieves high identification accuracy and requires little or no training [4,5,6]. In SSVEP- BCIs, the use of six or more channels of EEG signals from the occipital lobe has been proven to be sufficient to achieve excellent decoding performance [7]. Therefore, wearable EEG devices are considered suitable for the SSVEP paradigm, and they are used in many SSVEP-BCI studies. Zhu et al. used an eight-channel wearable SSVEP-BCI system to collect a dataset for developing decoding algorithms [8]. Na et al. designed an eight-channel wearable low-power EEG acquisition device for four-target SSVEP recognition [9]. On the other hand, some researchers paid attention to using few-channel EEGs for SSVEP decoding. Ge et al. designed a dual-frequency biased coding method and used a three-occipital-channel EEG to decode 48 targets with an accuracy of 76% in a 2 s time window [10], proving the availability of the SSVEP-BCI with few EEG channels. Moreover, several studies have shown that a single-channel EEG signal is feasible for few-target SSVEP detection [11,12,13,14,15]. It is clear that SSVEP identification based on few-channel EEGs is feasible in wearable BCIs.

As far as SSVEP identification is concerned, numerous algorithms have been developed [16,17]. Among them, canonical correlation analysis (CCA) is one of the mainstream fundamental approaches, which is free of training and determines the SSVEP target based on the correlation between a reference and the EEG signal [18]. On the basis of CCA, many variant algorithms have been developed, such as extended CCA (eCCA) [19] and filter bank CCA (FBCCA) [20]. Although traditional methods achieve good classification performance, the features extracted in these methods are relatively simple, which may not comprehensively represent EEG signals. In the last few years, deep learning technology has been rapidly employed in SSVEP decoding due to its capability to integrate feature extraction and classification. In particular, the convolutional neural network (CNN) is the most utilized neural network [21,22,23], as it offers advantages over other standard deep neural networks. A CNN was first applied to SSVEP identification by Cecotti et al. [24]. Nguyen et al. employed fast Fourier transform (FFT) to extract features from a single-channel EEG and then used a one-dimensional CNN to detect the SSVEP frequency [13]. Ravi et al. developed CNN models with the spectrum features derived from EEG signals as the input and found that the CNN based on complex spectrum features performed better than that based on magnitude spectrum features in the SSVEP-BCI [25]. Xing et al. constructed the frequency domain templates based on the prior knowledge of SSVEP and used a CNN for signal classification [26]. Zhao et al. fused the filter bank technique with a CNN to develop a filter bank CNN (FBCNN) based on the frequency domain SSVEP data [27]. Similarly, the combination of the filter bank technique and a CNN can be used for the analysis of time-domain SSVEP data [28]. Guney et al. proposed a deep neural network architecture consisting of 4 convolutional layers for processing time-domain EEG signals to predict SSVEP targets [29]. With the time-frequency sequences transformed from EEG signals, Li et al. developed a dilated shuffle CNN (DSCNN) to realize SSVEP classification [30]. In general, CNN-based methods tend to surpass the traditional methods. The convolutional layers in CNNs are considered to exploit the local spatial coherence inherent in SSVEP signals, making the models suitable for SSVEP analysis [31]. However, deep-learning-based methods typically require a lot of data for training and fine-tuning to achieve good results. In wearable BCIs, the reduction in data caused by the decreased EEG channels probably leads to a poor performance of the deep-learning-based method. Therefore, SSVEP identification based on few-channel EEGs remains challenging.

In order to implement the identification of SSVEP by using a few-channel EEG, a CNN-based decoding model is proposed in this study. The CNN-structure model is based on lightweight design to reduce the training data requirements associated with model complexity. In addition, considering the limited spatial information obtained from the few-channel signal, an attention mechanism is introduced to enhance the representation ability of spatial information of the model. Two SSVEP datasets were used for the method evaluation, including a 40-class dataset collected by a wearable EEG device and a public 12-class dataset collected by a conventional apparatus. Three-channel and single-channel data were applied for the comparison to evaluate the effectiveness of the proposed model on a few-channel EEG.

## 2. Materials and Methods

### 2.1. Datasets

#### 2.1.1. Dataset 1

In this study, a wearable ESPW308 EEG device (BlueBCI Ltd., Beijing, China) was used to collect an SSVEP dataset from six healthy subjects (three females and three males; average age: 25.33 ± 0.82 years) with normal or corrected-to-normal vision. This lightweight EEG apparatus is capable of acquiring eight-channel data from the occipital area (PO3, PO4, PO5, PO6, POz, O1, O2 and Oz). All subjects had no experience with SSVEP-BCIs before this experiment. The experiment was approved by the Institutional Review Board of the University of Hong Kong/Hospital Authority Hong Kong West Cluster.

A 40-target speller was used to induce the SSVEP, where the visual stimulation interface was a 4 × 10 flicker matrix displayed on a 24.5-inch LCD monitor with a full 1080 p resolution. Each flicker was presented as a 165 × 165-pixel square marked with a character. The flickers were encoded by the joint frequency and phase modulation (JFPM) method [32]. The frequency in Hz and phase in π for each stimulus is defined as follows:

(1) $$\left\{\begin{array}{l} f\left(i,j\right)=2i+0.2j+5.8\\ \varphi \left(i,j\right)=5i+0.5j-5.5 \end{array}\right.,$$

where $\left(i,j\right)$ represents the flicker located in the i-th row and j-th column.

Each subject completed 10 blocks of the experiment, with each block encompassing 40 trials that corresponded to all 40 targets. A trial contained a 1 s cue, a 3 s visual stimulation and a 1 s rest. It is noted that a strategy of simplifying the system setup was adopted in this experiment to reduce the experimental preparation time and enhance the practicality of the BCI in real-life applications [33]. As a result, the signal quality of this dataset might be degraded.

EEG signals from three occipital channels, O1, O2 and Oz, in Dataset 1 were selected as the three-channel EEG, and Oz was selected for the single-channel EEG.

#### 2.1.2. Dataset 2

To validate the effectiveness of the decoding model on EEG signals collected by a conventional device, a public SSVEP dataset was also used in this study. This dataset, which was presented by Nakanishi et al. [34], was collected by a BioSemi ActiveTwo EEG system (Biosemi Inc., Amsterdam, Netherlands) from ten healthy subjects (one female and nine males; average age: 28 years). With the acquisition system, eight-channel EEGs were recorded from the occipital area. During the experiment, a 12-target speller in the form of a 4 × 3 matrix was used. Each flicker was depicted as a 6 × 6-cm square on a 27-inch LCD monitor. The stimulus frequency in Hz and phase in π of each flicker were defined as follows:

(2) $$\left\{\begin{array}{l} f\left(i,j\right)=0.5i+2j+6.75\\ \varphi \left(i,j\right)=0.5i-0.5 \end{array}\right.,$$

where $\left(i,j\right)$ represents the flicker in the i-th row and j-th column.

Each subject performed a total of 15 experimental blocks, with each block consisting of 12 trials. A trial comprised a 1 s cue and a 4 s visual stimulation.

In Dataset 2, Oz and the two adjacent electrodes were selected for the three-channel EEG, and Oz was selected for the single-channel EEG.

### 2.2. SSVEP Identification

#### 2.2.1. Data Processing

For the original EEG signals, a fourth-order Butterworth filter is applied to remove noise and artifacts as much as possible. Due to the distinct features of SSVEP signals in the frequency domain, along with the automated feature extraction capabilities of neural networks, transforming time-domain signals into the frequency domain can enhance SSVEP identification [27]. Furthermore, deep learning models utilizing frequency-domain inputs generally have a relatively simple structure [31]. Therefore, the EEG time series is transformed into its frequency-domain counterpart through an FFT. Specifically, the time-domain signal is decomposed after the FFT as follows:

(3) FFTx=ReFFTx+jImFFTx,

where x is the input EEG data in the time domain.

Since FFT data have real and imaginary parts, a magnitude spectrum and a complex spectrum can be obtained depending on the combination of the real and imaginary parts. The magnitude spectrum retains the amplitude information of the Fourier spectrum and removes the phase information, while the complex spectrum retains both types of information. Previous studies have shown that CNNs using complex spectrum features as inputs outperform those based on magnetic spectrum features, as they can extract more discriminative information [25,27]. Therefore, the complex spectrum is used as the input in this study. Specifically, the real and imaginary parts of each channel are separated to form two vectors, which are then concatenated into a feature vector as the input for the neural network. Taking the three-channel EEG signal as an example, the complex FFT data are reconstructed as follows:

(4) I=ReFFTxO1,ImFFTxO1ReFFTxOz,ImFFTxOzReFFTxO2,ImFFTxO2,

where the real part is placed as the first half and the imaginary part is placed as the second half. This input is consistent in form with previous studies [25,27,35,36].

#### 2.2.2. Network Structure

**Figure 1:**
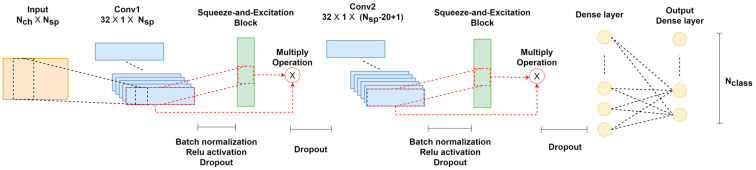
The architecture of atten-CCNN.

In this study, a CNN architecture called attention-based complex spectrum–CNN (atten-CCNN) is proposed, which integrates an attention mechanism with a CNN for SSVEP classification by using a few-channel EEG. The architecture of atten-CCNN is depicted in Figure 1, which was inspired by the complex spectrum–CNN (CCNN) model [25] and incorporates the attention mechanism from the squeeze-and-excitation (SE) network [37]. The atten-CCNN model consists of two stacked convolution–attention blocks for feature extraction, followed by a fully connected layer that performs non-linear transformation on the features and a dense layer with a softmax operation that is employed for classification. As for the convolution–attention block, a convolutional layer is followed by an activation operation, a batch normalization operation and a dropout operation. Then, a filter-wise attention layer is connected to the convolutional layer and a dropout operation. Additionally, an adjusted connection scheme is designed between the convolutional layer and attention layer.

Regarding the network hyperparameters, the input shape for atten-CCNN is denoted as $N_{ch}\times N_{sp}$, where $N_{ch}$ and $N_{sp}$ are the dimensions of the complex FFT data. The first convolutional layer, Conv1, with a kernel size of [32 × 1] calculates the contribution weight among the selected EEG channels. The second convolutional layer, Conv2, performs spectral-level representation and has a fixed kernel size of [1 × 20]. It is worth noting that Conv1 uses the “valid” padding mode while Conv2 uses the “same” padding mode to help reduce the model complexity while preserving the learned convolutional features. Both convolutional layers have 32 filters, providing sufficient power for feature extraction while keeping the number of network parameters relatively low. The first dense layer consists of 144 neurons with the “ReLU” activation function. The bottom dense layer applies linear transformation to the features, and a softmax operation is used with an output shape that corresponds to the number of targets in the SSVEP dataset.

#### 2.2.3. Filter-Wise Attention Mechanism

**Figure 2:**
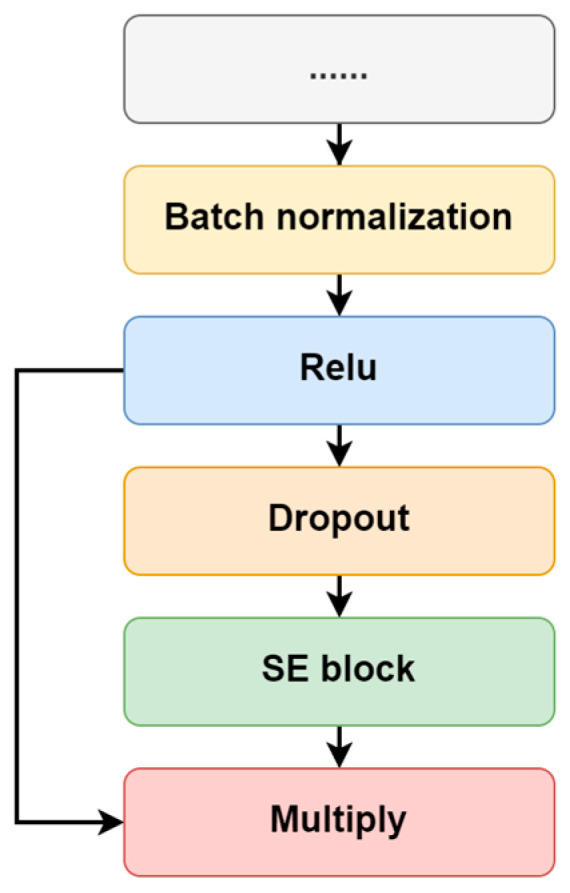
The connection between the convolutional layer and SE attention.

As the key component of atten-CCNN, the attention mechanism is employed to increase the network’s representation space by reweighting the contribution of different feature maps (filters) in each convolutional layer. Specifically, the SE block is used in this study. Owing to the lightweight structure, the SE block slightly increases the computational load and complexity of the original models. Moreover, the SE block has high compatibility and can be integrated into existing network architectures without major modifications. In terms of the connection between the SE attention with the convolutional layer, as shown in Figure 2, an adjusted connection scheme was designed rather than simply using SE block as a plug-and-play module. In this design, the key vector used for calculating the attention vector is derived from the dropout-passed feature maps of the last convolutional layer, while the feature maps that do not undergo dropout are used to compute the final attention-enhanced data. This structural design retains the information from the original feature maps while mitigating overfitting during attention weight calculation.

#### 2.2.4. Training Hypermeters

In this study, both subject-independent and subject-dependent strategies were tested on two datasets. For the subject-independent strategy, leave-one-person-out cross-validation was used. If a dataset contained n subjects, the model underwent training utilizing the data of n−1 subjects and was subsequently evaluated using the data from the remaining subject. In order to implement the model training and testing, all the EEG data were split into non-overlapping segments according to the data length being tested. The finial parameters for the model were as follows: learn rate (0.001), dropout ratio (0.25), L2 regularization (0.001), number of epochs (120) and batch size (256).

For the subject-dependent strategy, 10-fold cross-validation was performed on each subject’s data. The EEG data of a subject were firstly segmented into non-overlapping segments, and then the segments were divided into 10 sets randomly. Training data were formed by taking nine sets and leaving one set out for testing. Except for the batch size (16), the other parameters were the same as those in the subject-independent strategy. Additionally, SGD was selected as the optimizer for all the models in all the training strategy modes.

### 2.3. Performance Evaluation

#### 2.3.1. Baseline Methods

To verify the effectiveness of the proposed atten-CCNN model, CCNN [25], EEGNet [38] and SSVEPformer [36] were used as the baseline models for comparison in this study.

CCNN is mainly composed of two convolutional layers in series, using the complex spectrum of EEG signal as the input. The first convolutional layer is used for spatial filtering and operates on the channels of the input, while the second convolutional layer is for temporal filtering and extracts features along the frequency components. Both convolutional layers are followed by a batch normalization layer, ReLU activation layer and dropout layer. Finally, a fully connected layer is employed in CCNN for classification.

EEGNet is a popular CNN-based architecture for EEG decoding, and it takes time-domain data as the input. EEGNet adopts a four-layer compact structure. The first layer is a convolution layer, which simulates band-pass filtering on each channel. Next is a spatial filtering layer to weight the data through depth-wise convolution. The third layer is a separate convolutional layer to extract category information. The last layer is a fully connected layer for classification.

SSVEPformer is one of the state-of-the-art models for SSVEP identification, which also takes the complex spectrum as the input and consists of three core components: channel combination, SSVEPformer encoder and multilayer perceptron (MLP) head. Firstly, the channel combination block performs weighted combinations of the input through convolutional layers. Then, the SSVEPformer encoder utilizes two sequential sub-encoders to extract features, each of which includes a CNN and a channel MLP. At last, the MLP head block uses two fully connected layers to implement classification.

#### 2.3.2. Metrics

Two metrics were employed to assess the effectiveness of the models, including classification accuracy and information transfer rate (ITR). Accuracy is defined as the proportion of trials where the model makes a correct identification. ITR is a frequently-used parameter for evaluating BCI performance, which is estimated as follows:

(5) $$ITR=60\left(\log_{2}K+P\log_{2}P+\left(1-P\right)\log_{2}\left(\frac{1-P}{K-1}\right)\right)/T,$$

where K is the number of targets, P is the classification accuracy and T is the target selection time. In addition to the data length of the SSVEP signal, the target selection time included a gaze shift time in this study to imitate the actual use of the BCI. The gaze shift time was set to 0.55 s according to Chen et al.’s study [20].

## 3. Results

### 3.1. Dataset 1

**Figure 3:**
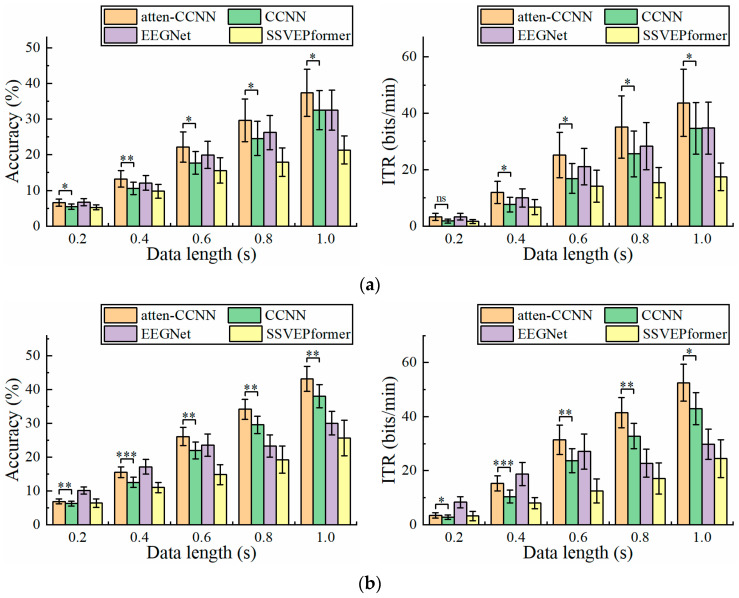
The accuracy and ITR on Dataset 1 with three-channel EEG under (**a**) subject-independent and (**b**) subject-dependent conditions. The error bars represent the standard error of mean (SEM). The significance of the difference between atten-CCNN and CCNN is marked by ns (*p* ≥ 0.05), * (*p* < 0.05), ** (*p* < 0.01) or *** (*p* < 0.001).

Figure 3 illustrates the classification accuracy and ITR on the 40-class wearable SSVEP dataset with a three-channel EEG achieved by the four decoding models. Furthermore, the paired t test was employed to ascertain the difference in the metrics between atten-CCNN and CCNN to verify the improvement in the proposed method. The results show that under both the subject-independent and subject-dependent conditions, atten-CCNN performed better than CCNN with a significant difference at all data lengths. Moreover, the advantage of atten-CCNN over CCNN basically expanded as the data length of the EEG signal increased. At a data length of 1 s, the subject-independence accuracy of atten-CCNN was 37.38%, which was 4.86% ahead of CCNN, and its subject-dependence accuracy was 43.21%, leading CCNN by 5.17%. Compared with subject-independent strategies, deep learning models generally achieve better performances under subject-dependent strategies [25,27,39,40]. This finding was consistent in this study. As for EEGNet, it performed well on the short-time EEG signals in this dataset, although it was found to be not as good as a CCNN in previous study [41]. Indeed, EEGNet outperformed atten-CCNN and CCNN at 0.2 s. But when the data length exceeded 0.4 s, atten-CCNN surpassed EEGNet in both strategies, and their gap enlarged with the data length. For SSVEPformer, it seems unsuitable for this dataset as it performed the worst among the four models regardless of the training strategies.

**Figure 4:**
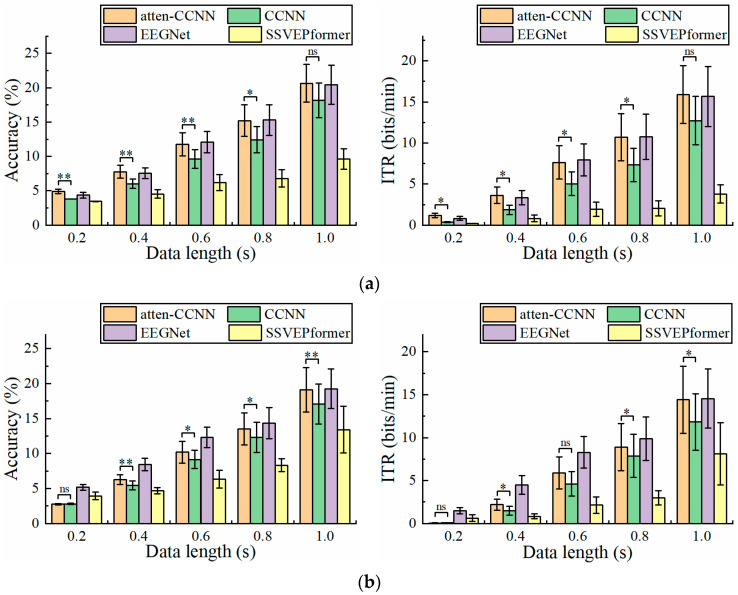
The accuracy and ITR on Dataset 1 with single-channel EEG under (**a**) subject-independent and (**b**) subject-dependent conditions. The error bars represent SEM, and the significance of the difference between atten-CCNN and CCNN is marked by ns (*p* ≥ 0.05), * (*p* < 0.05), ** (*p* < 0.01) or *** (*p* < 0.001).

The performance of the models on Dataset 1 when using a single-channel EEG for decoding is shown in Figure 4. The relationship between atten-CCNN and CCNN when using the single-channel EEG was similar to that with the three-channel EEG. For the long-time EEG signals in this study, the advantages of atten-CCNN tended to be more obvious. However, there was no significant difference between atten-CCNN and CCNN at 1 s under the subject-independent conditions, which seems to indicate that the large difference at this time point was mainly caused by individual subjects. On the other hand, the results show that EEGNet performed well in single-channel EEG decoding. In the subject-independent situation, EEGNet and atten-CNN performed similarly at various data lengths, outperforming CCNN. In the subject-dependent scenario, EEGNet performed the best among the four models on the short-time signal, and atten-CCNN achieved the same performance as EEGNet at 1 s. But for SSVEPformer, it did not demonstrate the superiority in the single-channel EEG decoding.

### 3.2. Dataset 2

**Figure 5:**
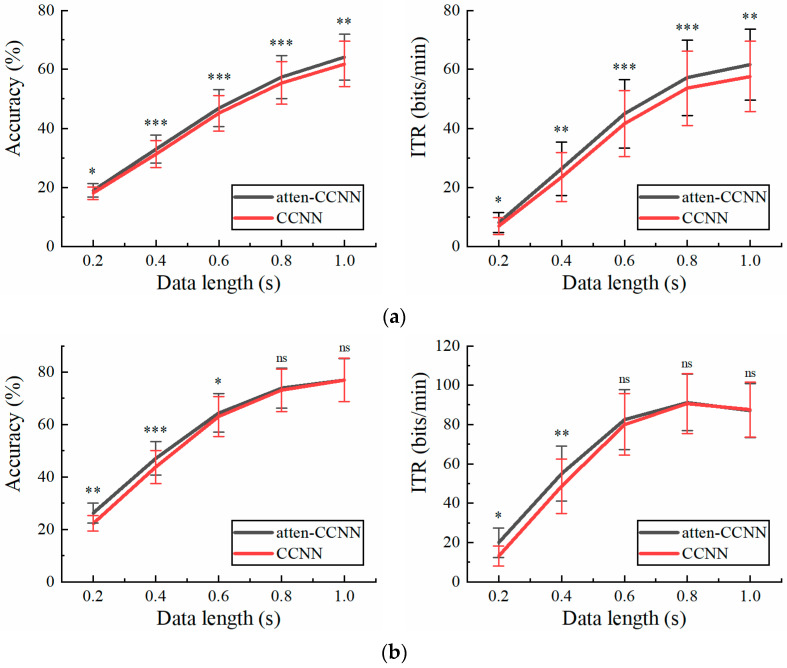
The accuracy and ITR on Dataset 2 with three-channel EEG under (**a**) subject-independent and (**b**) subject-dependent conditions. The error bars represent SEM, and the significance of the difference is marked by ns (*p* ≥ 0.05), * (*p* < 0.05), ** (*p* < 0.01) or *** (*p* < 0.001).

Since CCNN, EEGNet and SSVEPformer have been tested and compared on Dataset 2 in previous studies [36,42,43], only atten-CCNN and CCNN were used for comparison in this part to emphasize the changes in the performance of the proposed model relative to its original model. Figure 5 illustrates the performance of atten-CCNN and CCNN on Dataset 2 with a three-channel EEG. Intuitively, the performance difference between atten-CCNN and CCNN showed different trends under the two conditions. Under the subject-independent conditions, the advantage of atten-CCNN expanded as the data length increased, and the gap between them was significant at all data lengths. However, in the subject-dependent case, atten-CCNN only had advantages on the short-time data. When the time window was greater than 0.6 s, the improvement in atten-CCNN was not significant. In terms of the best decoding performance with the three-channel EEG, atten-CCNN achieved a subject-independent accuracy of 64.17% at 1 s, which was 2.35% higher than CCNN, and the highest ITR was 61.55 bits/min at 1 s. On the contrary, under the subject-dependent conditions, the decoding models achieved the maximum ITR at a data length of 0.8 s, and the result of atten-CCNN was 91.20 bits/min.

**Figure 6:**
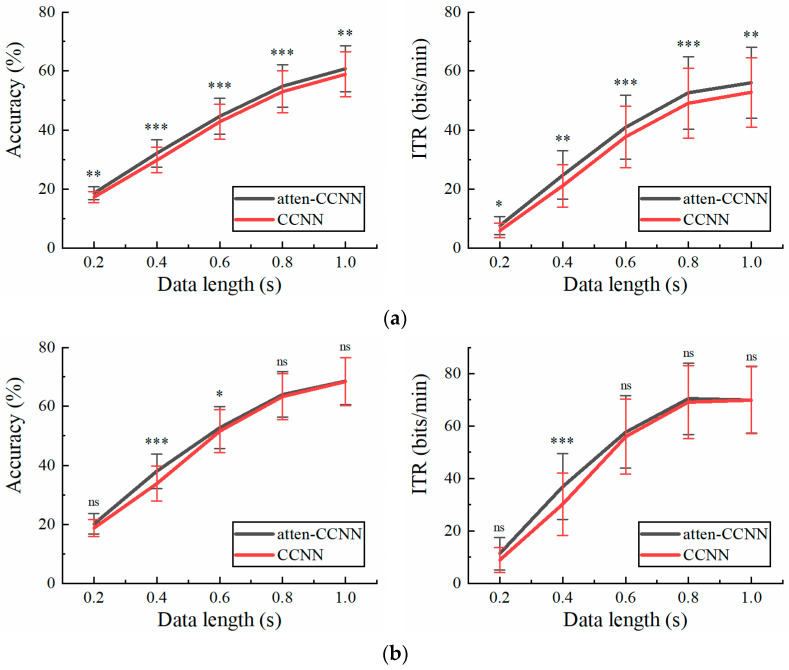
The accuracy and ITR on Dataset 2 with single-channel EEG under (**a**) subject-independent and (**b**) subject-dependent conditions. The error bars represent SEM, and the significance of the difference is marked by ns (*p* ≥ 0.05), * (*p* < 0.05), ** (*p* < 0.01) or *** (*p* < 0.001).

Figure 6 illustrates the accuracy and ITR of the two models with a single-channel EEG on Dataset 2 under the two conditions. Clearly, the testing strategy had a great impact on the performance of the models in the single-channel EEG decoding. Under the subject-independent conditions, atten-CCNN outperformed CCNN at all data lengths. The largest accuracy difference between two models occurred at 0.4 s, which was 2.24%. In contrast, in the subject-dependent situation, the new model only significantly outperformed CCNN at 0.4 s, with an accuracy improvement of 4.20%.

Comparing the decoding performance of the models on the two datasets, it was observed that the subject-independent improvement brought by atten-CNN was broadened with the increase in the data length. The improvement in atten-CNN was numerically greater on the wearable dataset. But under the subject-dependent conditions, the two datasets presented different results. Atten-CNN also showed a greater improvement over time on Dataset 1, while its effective improvement on Dataset 2 only occurred in a short time window, such as 0.4 s.

### 3.3. Effect of Number of EEG Channels

**Figure 7:**
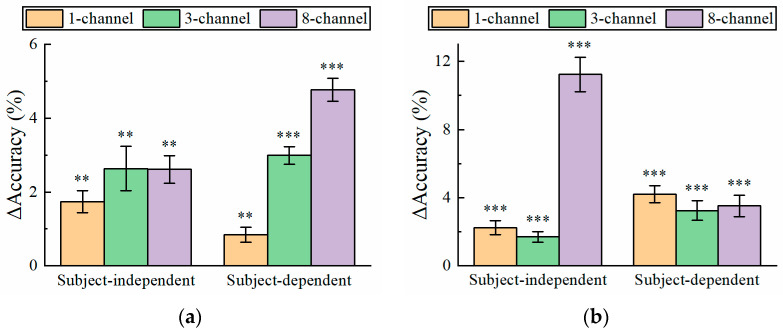
The accuracy improvement in atten-CCNN over CCNN on (**a**) Dataset 1 and (**b**) Dataset 2 when the data length was 0.4 s. The error bars represent SEM, and the significance of the difference between two models is marked by ns (*p* ≥ 0.05), * (*p* < 0.05), ** (*p* < 0.01) or *** (*p* < 0.001).

It can be found from Figure 3, Figure 4, Figure 5 and Figure 6 that for the short-time EEG signals, the improvement in atten-CCNN relative to CCNN was significant on both datasets, whether in the subject-independent or subject-dependent strategies. In order to further explore the relationship between the improvement in the proposed model and the number of EEG channels, the decoding results on a few-channel EEG were compared with that on eight-channel data because eight-channel EEGs are considered sufficient for multi-target SSVEP identification. Figure 7 shows the accuracy difference between atten-CCNN and CCNN when the data length was 0.4 s. Obviously, whether a single-channel, three-channel or eight-channel EEG was used for decoding, the accuracy improvement in atten-CCNN compared to CCNN was significant. According to Figure 7, the magnitude of the improvement in atten-CCNN did not appear to be strongly related to the number of EEG channels. There is no doubt that the algorithms were generally more effective as the number of channels increased. Nevertheless, the proposed model still maintained and even enlarges its advantage as the number of channels increased.

## 4. Discussion

A CNN-structure model named atten-CCNN was developed by fusing an SE block in a complex-spectrum CNN. Plenty of studies have shown that excellent SSVEP identification can be achieved by decoding many EEG channels. However, wearable BCIs equipped with a large quantity of electrodes are not appropriate and feasible in real-life applications. Therefore, we evaluated the performance of the classification methods in this study with a few-channel EEG. Similarly, although long signal segments substantially elevated the classification accuracy of the algorithm, the ITR generally decreased. Therefore, we selected EEG segments within 1 s for evaluation. The results show that atten-CCNN outperformed the baseline methods on both the wearable SSVEP dataset and conventional dataset in both subject-independent and subject-dependent scenarios.

CCNN was chosen as the backbone of the model developed in this study because of its simplicity and scalability. It has the flexibility to modify the network structure. We increased the filters in the convolutional layer to extract various types of information from the EEG data, which is also suitable for the filter-level attention mechanism. Additionally, we added a fully connected layer after the feature flattening to further facilitate the learning capacity of the model. As the key difference between the new model and the original CCNN, we added an SE block after each convolutional layer. SE blocks are commonly used attention modules, which are used in conjunction with existing models to improve the performance by concentrating on essential features while restraining non-essential ones. Since their emergence, SE blocks have been used in EEG analysis under different tasks [44,45,46,47]. In the conventional convolutional layer with multiple spatial filters, each spatial filter uses a local receptive field to avoid the output being affected by contextual information outside that region. In order to leverage the information beyond the local receptive field, the squeeze part in the SE block utilizes global average pooling to produce channel-wise statistics. Then, the excitation part in the SE block applies a gate mechanism consisting of two fully connected layers to learn the nonlinear relationship between the channels, thereby exploiting the information obtained in the squeeze operation and completely capturing channel-wise dependencies. Finally, the SE block takes the weight output by the excitation operation as the importance of channels and completes the recalibration of the original features by reweighting the features of each channel, thus boosting feature discriminability and improving the network’s performance. The experiments in this study demonstrate that the SE block could improve the performance of the CNN in SSVEP identification with a few-channel EEG. However, SSVEPformer, which also involves an attention mechanism, performed poorly in this study. A potential factor contributing to this result may be its inherently complex network architecture, which may not be suitable for the analysis of few-channel data. Although SSVEPformer has a channel combination block designed to process multi-channel EEGs, this block may not have the expected effect when dealing with few-channel data. Similarly, a few-channel signal may affect the feature extraction ability of the networks. Based on the experimental results, this study verified the hypothesis in Chen et al.’s study that a limited amount of data is a major challenge for SSVEPformer to maintain good performance [36]. In contrast, the results show that the compact EEGNet performed well on small-dimensional data, especially short-time, single-channel EEGs, demonstrating its potential in single-channel decoding. On the other hand, it was found that the improvement in atten-CCNN relative to the original model on Dataset 1 was more noticeable than that on Dataset 2. This may be due to the difference in the signal quality between the two datasets. As Dataset 1 was collected by a wearable EEG device under a simplified system setup, the signal quality of Dataset 1 is lower than that of Dataset 2. The noise mixed in the EEG signal interferes with the feature extraction ability of the convolutional layer, but the SE block has the function of strengthening important features and weakening noise or unimportant features. Therefore, we believe that the atten-CCNN model can perform well even for EEG signals with a low signal-to-noise ratio.

The decoding performance on a three-channel EEG and a single-channel EEG was compared in this study. There is no doubt that the three-channel results were better than the single-channel results. But compared with the 12-class dataset, the gap between them was much larger on the 40-class dataset. In the case of a large number of targets, a single-channel EEG seems to be incompetent for SSVEP identification, especially at short time windows. As for the three-channel EEG with a reasonable data length, it can cope with the recognition of a mass of targets. For fewer targets, such as 12 targets, a single-channel EEG is an attractive option as the gap between three-channel EEG decoding and single-channel decoding is not particularly large. Owing to the improved decoding capabilities brought by deep learning, SSVEP-BCIs based on few-channel EEGs are becoming feasible and practical in daily-life applications. In addition, although the proposed atten-CCNN was designed for few-channel EEG decoding, its improvement over the baseline model was more considerable when the number of EEG channels increased, as shown in Figure 7, indicating the adaptability of this model to EEG decoding with different channel numbers.

There are several limitations in this study. Two SSVEP datasets were used to verify the performance of the models in this study, but the wearable dataset only involved six subjects. Although the statistical analysis demonstrated the effectiveness of the proposed model, a small sample size may lead to great uncertainty that can affect the credibility of the results. Therefore, we plan to collect more data to further validate the model. Secondly, for the input of the classification model, the most common form of complex spectrum was used in this study. Indeed, there are other ways of composing the real and imaginary parts, such as placing them on different rows of the vector [48]. In the next step, we will compare different forms of model inputs to determine a suitable one. On the other hand, the decoding model was enhanced by combining multiple modules in this study. In SSVEP recognition, the filter bank technique is proven to be a simple and effective strategy to enhance decoding methods, both for traditional methods [20,49] and deep learning methods [27,28,43,48,50], because the filter bank technique takes advantage of the harmonic characteristics of SSVEP. It is believed that the filter bank technique will have similar effects on atten-CCNN, so we plan to apply this technique to atten-CCNN to further improve its performance. Overall, although the new model was improved compared to the baseline models, there is room for improvement in this model.

## 5. Conclusions

This study introduces an atten-CCNN model for SSVEP identification with few-channel EEGs, which takes the complex spectrum of the EEG signal as the input and integrates an SE block with a CNN. The proposed method was evaluated by a wearable SSVEP dataset and a public dataset under subject-independent and subject-dependent conditions. The results show that, whether for a three-channel or single-channel EEG, the new model had better performance than the baseline models. The improvement in the BCI performance demonstrates the efficacy of incorporating attention mechanisms to bolster the decoding ability of CNNs on few-channel EEGs. The SSVEP identification algorithm based on a few-channel EEG is particularly suitable for wearable BCIs as it achieves good performance with limited information. We believe that this decoding method, combined with the natural advantages of wearable BCIs, can promote the application of BCIs in real life.

## Data Availability

The public dataset can be downloaded from the following link: https://github.com/mnakanishi/12JFPM_SSVEP (accessed on 20 October 2023). The experimental dataset collected in this study is available from the corresponding author upon reasonable request, which is not publicly available due to privacy.
